# Supplementary material for: The importance of individuals of different sizes in the population maintenance of a palm species used by the Fulni-ô Indigenous People in northeast Brazil
Source: PeerJ. 2025 Aug 13;13:e19739. doi: 10.7717/peerj.19739 (PMC12357544; doi:10.7717/peerj.19739)
Supplement: Supplemental Information 3 — The selected models are in bold with their respective coefficients. [file peerj-13-19739-s003.docx]

| Table S2. Survival model structures based on plant size (height) with corresponding Akaike Information Criterion (AIC) scores, R², p-values, residual standard errors, and likelihood values for three populations of the palm *Syagrus coronata* across three sampling intervals in Águas Belas, Pernambuco, northeastern Brazil. | | | | | | | | |
| --- | --- | --- | --- | --- | --- | --- | --- | --- |
| Sampling Interval 1 | | | | | | | | |
| Population | Model Structure | AIC | | R² | p | | Residual standard error | Likelihood |
| Low | surv ~ 1 | 21.0 | | - | - | | 0.1516 | 40.7147 |
|  | **surv ~ 5.53size** | **21.0** | | 0.1883107 | 0.1883107 | | 0.1503 | 41.96288 |
|  | surv ~ -0.47size -12.99size² | 22.7 | | 0.1943814 | 0.1943814 | | 0.1503 | 42.02395 |
|  | surv ~ -6.10size + 44.31size² - 40.37size³ | 24.6 | | 0.2273600 | 0.2273600 | | 0.1503 | 42.1682 |
|  | | | | | | | |  |
| Sampling Interval 2 | | | | | | | | |
|  | Model Structure | AIC | | R² | p | | Residual standard error | Likelihood |
| Low | surv ~ 1 | 45.5 | | **-** | **-** | | 0.2562 | -4.427649 |
|  | surv ~ 9.43size | 25.7 | | 0.04104716 | 0.0001029 | | 0.1965 | 18.50017 |
|  | surv ~ 12.74size -6.12size² | 27.5 | | 0.49297217 | 0.0001029 | | 0.1965 | 24.13073 |
|  | **surv ~ 19053.8size -95306.41size² + 119598.4size³** | **17.5** | | 0.49297219 | 0.0001029 | | 0.1965 | 29.01 |
|  | | | | | | | |  |
| Sampling Interval 3 | | | | | | | | |
|  | Model Structure | AIC | | R² | p | | Residual standard error | Likelihood |
| Low | surv ~ 1 | 70.3 | | - | - | | 0.3556 | -31.07146 |
|  | surv ~ 2.71size | 70.9 | | 0.02200212 | 0.2377 | | 0.3547 | -30.35247 |
|  | **surv ~ 26.59size -26.43size²** | **67.8** | | 0.09515525 | 0.2377 | | 0.3547 | -26.34891 |
|  | surv ~ -4.12size + 50.95size² - 58.11size³ | 68.9 | | 0.11122350 | 0.2377 | | 0.3547 | -26.01341 |
|  | | | | | | | |  |
| Sampling Interval 1 | | | | | | | | |
| Intermediate | Model Structure | AIC | | R² | p | | Residual standard error | Likelihood |
|  | surv ~ 1 | 168.6 | | - | - | | 0.322 | -65.48833 |
|  | surv ~ 2.22size | 142.9 | | 0.3075434 | 7.063e-08 | | 0.3028 | -50.80617 |
|  | **surv ~ 7.36size +8.89size²** | **119.9** | | 0.6768398 | 7.063e-08 | | 0.3028 | -48.46614 |
|  | surv ~ 10.69size + 22.60size² + 11.36size³ | 119.6 | | 0.6768400 | 7.063e-08 | | 0.3028 | -41.18602 |
|  | | | | | | | |  |
| Sampling Interval 2 | | | | | | | | |
|  | Model Structure | | AIC | R² | | p | Residual standard error | Likelihood |
| Intermediate | surv ~ 1 | | 257.7 | **-** | | **-** | 0.4243 | -131.482 |
|  | **surv ~ 3.69size** | | **156.4** | 0.01285018 | | < 2.2e-16 | 0.3301 | -71.98373 |
|  | surv ~ 4.37size +1.59size² | | 156.3 | 0.05613830 | | < 2.2e-16 | 0.3301 | -69.38761 |
|  | surv ~ 5.20size -0.56size² - 3.05size³ | | 156 | 0.09514895 | | < 2.2e-16 | 0.3301 | -65.20029 |
|  | | | | | | | |  |
| Sampling Interval 3 | | | | | | | | |
| Intermediate | Model Structure | | AIC | R² | | p | Residual standard error | Likelihood |
|  | surv ~ 1 | | 229.5 | **-** | | - | 0.4515 | -118.6392 |
|  | **surv ~ 3.54size** | | **155.3** | 7.996414e-05 | | < 2.2e-16 | 0.3551 | -72.27304 |
|  | surv ~ 3.91size – 1.73size² | | 155.6 | 1.372488e-02 | | < 2.2e-16 | 0.3551 | -71.26376 |
|  | surv ~ size + size² + size³ | | 154.5 | 2.827056e-02 | | < 2.2e-16 | 0.3551 | -64.95492 |
| Samplig Interval 1 | | | | | | | | |
| High | Model Structure | | AIC | R² | | p | Residual standard error | Likelihood |
|  | surv ~ 1 | | 131.6 | **-** | | **-** | 0.341 | -56.08954 |
|  | surv ~ 1.70size | | 121.1 | 0.01705142 | | 0.0001101 | 0.3266 | -48.49719 |
|  | **surv ~ 0.89size -3.96size²** | | **112.6** | 0.13602693 | | 0.0001101 | 0.3266 | -38.54191 |
|  | surv ~ 2.30size -4.50size² -3.07size³ | | 112.5 | 0.18866695 | | 0.0001101 | 0.3266 | -38.17307 |
|  | | | | | | | |  |
| Samplig Interval 2 | | | | | | | | |
| High | Model Structure | | AIC | R² | | p | Residual standard error | Likelihood |
|  | surv ~ 1 | | 158.0 | **-** | | **-** | 0.3994 | -78.15888 |
|  | surv ~ 2.99size | | 119.2 | 0.01285018 | | 5.565e-13 | 0.3386 | -51.74341 |
|  | **surv ~ 3.88size +3.53size²** | | **112.6** | 0.05613830 | | 5.565e-13 | 0.3386 | 50.39469 |
|  | surv ~ 4.02size + 3.17size² - 0.54size³ | | 114.6 | 0.09514895 | | 5.565e-13 | 0.3386 | -46.92011 |
|  | | | | | | | |  |
| Samplig Interval 3 | | | | | | | | |
| High | Model Structure | | AIC | R² | | p | Residual standard error | Likelihood |
|  | surv ~ 1 | | 114.0 | - | | - | 0.3611 | -51.92479 |
|  | **surv ~ 2.90size** | | **88.6** | 0.004721262 | | 3.319e-10 | 0.3108 | -31.80433 |
|  | surv ~ 2.88size -2.02size² | | 89.5 | 0.060107616 | | 3.319e-10 | 0.3108 | -28.71295 |
|  | surv ~ 5.50size -1.94size² -6.81size³ | | 87.3 | 0.096515661 | | 3.319e-10 | 0.3108 | -25.70651 |
