# Supplementary material for: The importance of individuals of different sizes in the population maintenance of a palm species used by the Fulni-ô Indigenous People in northeast Brazil
Source: PeerJ. 2025 Aug 13;13:e19739. doi: 10.7717/peerj.19739 (PMC12357544; doi:10.7717/peerj.19739)
Supplement: Supplemental Information 4 — The selected models are in bold with their respective coefficients. [file peerj-13-19739-s004.docx]

| Table S3. Growth model structures based on plant size (height) with corresponding Akaike Information Criterion (AIC) scores, R², p-values, residual standard errors, and likelihood values for three populations of the palm *Syagrus coronata* across three sampling intervals in Águas Belas, Pernambuco, northeastern Brazil. | | | | | | |
| --- | --- | --- | --- | --- | --- | --- |
| Sampling Interval 1 | | | | | | |
| Population | Model Structure | AIC | R² | p | Residual standard error | Likelihood |
| Low | sizeNext ~ 1 | -78.0 | - | - | 0.2384 | 1.775162 |
|  | sizeNext ~ 0.94size | -332.4 | 0.9527539 | < 2.2e-16 | 0.03267 | 169.2149 |
|  | **sizeNext ~ 1.11size -0.24size²** | **-336.2** | 0.9558648 | < 2.2e-16 | 0.03267 | 169.2149 |
|  | sizeNext ~ 1.25size -0.80size² + 0.58size³ | -335.2 | 0.9564047 | < 2.2e-16 | 0.03267 | 169.2149 |
|  | | | | | |  |
| Sampling Interval 2 | | | | | | |
|  | Model Structure | AIC | R² | p | Residual standard error | Likelihood |
| Low | sizeNext ~ 1 | -101.4 | - | - | 0.1587 | 35.06662 |
|  | sizeNext ~ 0.92size | -335.9 | 0.9479864 | < 2.2e-16 | 0.02892 | 170.9461 |
|  | **sizeNext ~ 1.18size -0.29size²** | **-339.7** | 0.9516105 | < 2.2e-16 | 0.02892 | 170.9461 |
|  | sizeNext ~ 1.45size -1.03size² + 0.59size³ | -338.4 | 0.9520709 | < 2.2e-16 | 0.02892 | 170.9461 |
|  | | | | | |  |
| Sampling Interval 3 | | | | | | |
|  | Model Structure | AIC | R² | p | Residual standard error | Likelihood |
| Low | sizeNext ~ 1 | -81.1 | - | - | 0.1685 | 26.5745 |
|  | **sizeNext ~ 1.00size** | **-158.3** | 0.6772831 | < 2.2e-16 | 0.07592 | 82.1554 |
|  | sizeNext ~ 1.78size~-0.78size² | -158.8 | 0.6886097 | < 2.2e-16 | 0.07592 | 82.1554 |
|  | sizeNext ~ -0.07size + 3.48size² - 3.05size³ | -157.8 | 0.6927825 | < 2.2e-16 | 0.07592 | 82.1554 |
|  | | | | | |  |
| Sampling Interval 1 | | | | | | |
| Intermediate | Model Structure | AIC | R² | p | Residual standard error | Likelihood |
|  | sizeNext ~ 1 | 300.5 | - | - | 0.5507 | -192.7439 |
|  | sizeNext ~ 0.95size | -325.7 | 0.9540123 | < 2.2e-16 | 0.1078 | 165.8712 |
|  | sizeNext ~ 0.96size~0.07size² | -328.8 | 0.9551374 | < 2.2e-16 | 0.1078 | 165.8712 |
|  | **sizeNext ~ 1.07size + 0.024size² -0.225size³** | **-343.8** | 0.9587220 | < 2.2e-16 | 0.1078 | 165.8712 |
|  | | | | | |  |
| Sampling Interval 2 | | | | | | |
|  | Model Structure | AIC | R² | p | Residual standard error | Likelihood |
| Intermediate | sizeNext ~ 1 | 189.7 | **-** | **-** | 0.4666 | -124.9257 |
|  | sizeNext ~ 0.87size | -361.6 | 0.9537716 | < 2.2e-16 | 0.08764 | 183.8125 |
|  | sizeNext ~ 0.86size~0.14size² | -383.3 | 0.9594713 | < 2.2e-16 | 0.08764 | 183.8125 |
|  | **sizeNext ~ 0.96size + 0.17size² -0.23size³** | **-395.5** | 0.9625423 | < 2.2e-16 | 0.08764 | 183.8125 |
|  | | | | | |  |
| Sampling Interval 3 | | | | | | |
| Intermediate | Model Structure | AIC | R² | p | Residual standard error | Likelihood |
|  | sizeNext ~ 1 | 114.6 | **-** | - | 0.3754 | -60.56126 |
|  | **sizeNext ~ 1.02size** | **-47.7** | 0.6986859 | < 2.2e-16 | 0.2003 | 26.87235 |
|  | sizeNext ~ 1.09size-0.14size² | -47.1 | 0.7017304 | < 2.2e-16 | 0.2003 | 26.87235 |
|  | sizeNext ~ 1.11size -0.09size² -0.09size³ | -45.2 | 0.7018678 | < 2.2e-16 | 0.2003 | 26.87235 |
|  | | | | | |  |
| Samplig Interval 1 | | | | | | |
| High | Model Structure | AIC | R² | p | Residual standard error | Likelihood |
|  | sizeNext ~ 1 | 100.4 | **-** | **-** | 0.4313 | -90.22956 |
|  | sizeNext ~ 0.93size | -412.7 | 0.9727289 | < 2.2e-16 | 0.05637 | 209.3342 |
|  | sizeNext ~ 0.93size~0.08size² | -418.5 | 0.9741836 | < 2.2e-16 | 0.05637 | 209.3342 |
|  | **sizeNext ~ 1.03size + 0.07size² -0.27size³** | **-433** | 0.9769915 | < 2.2e-16 | 0.05637 | 209.3342 |
|  | | | | | |  |
| Samplig Interval 2 | | | | | | |
| High | Model Structure | AIC | R² | p | Residual standard error | Likelihood |
|  | sizeNext ~ 1 | 73.0 | **-** | **-** | 0.3852 | -60.39675 |
|  | sizeNext ~ 0.89size | -430.9 | 0.9537716 | < 2.2e-16 | 0.04309 | 218.427 |
|  | sizeNext ~ 0.89size~0.08size² | -441.5 | 0.9594713 | < 2.2e-16 | 0.04309 | 218.427 |
|  | **sizeNext ~ 1.00size + 0.10size² -0.26size³** | **-471.8** | 0.9625423 | < 2.2e-16 | 0.04309 | 218.427 |
|  | | | | | |  |
| Samplig Interval 3 | | | | | | |
| High | Model Structure | AIC | R² | p | Residual standard error | Likelihood |
|  | sizeNext ~ 1 | 25.2 | - | - | 0.3639 | -47.23106 |
|  | **sizeNext ~ 0.94size** | **-264.3** | 0.9275892 | < 2.2e-16 | 0.07228 | 135.1327 |
|  | sizeNext ~ 0.94size-0.00size² | -262.2 | 0.9275893 | < 2.2e-16 | 0.07228 | 135.1327 |
|  | sizeNext ~ 0.97size + 0.02size² -0.07size³ | -260.5 | 0.9277529 | < 2.2e-16 | 0.07228 | 135.1327 |
