# Supplementary material for: The importance of individuals of different sizes in the population maintenance of a palm species used by the Fulni-ô Indigenous People in northeast Brazil
Source: PeerJ. 2025 Aug 13;13:e19739. doi: 10.7717/peerj.19739 (PMC12357544; doi:10.7717/peerj.19739)
Supplement: Supplemental Information 5 [file peerj-13-19739-s005.docx]

**Table S4.** Reproductive individuals of *Syagrus coronata* per population (under low, intermediate, and high harvest frequencies) in year t, seedling recruitment in year t+1, and fertility values (f(x,y)) per sampling interval in Águas Belas, Pernambuco, northeast Brazil.

| Population | Sampling Interval | Number of seedlings in t+1 | Number of reproductive individuals in t | Fertility f(x,y) |
| --- | --- | --- | --- | --- |
| Low | 1 | 2 | 60 | 0.03 |
|  | 2 | 2 | 60 | 0.03 |
|  | 3 | 2 | 59 | 0.03 |
| Intermediate | 1 | 31 | 68 | 0.45 |
|  | 2 | 11 | 68 | 0.16 |
|  | 3 | 1 | 67 | 0.01 |
| High | 1 | 14 | 76 | 0.18 |
|  | 2 | 5 | 74 | 0.06 |
|  | 3 | 6 | 71 | 0.28 |
